# Recurrent event survival analysis predicts future risk of hospitalization in patients with paroxysmal and persistent atrial fibrillation

**DOI:** 10.1371/journal.pone.0217983

**Published:** 2019-06-07

**Authors:** Jakob Schroder, Olivier Bouaziz, Bue Ross Agner, Torben Martinussen, Per Lav Madsen, Dana Li, Ulrik Dixen

**Affiliations:** 1 Department of Cardiology, Bispebjerg University Hospital, Copenhagen, Denmark; 2 Laboratory MAP5, University Paris Descartes and CNRS, Sorbonne Paris Cité, Paris, France; 3 Department of Cardiology, Hvidovre University Hospital, Copenhagen, Denmark; 4 Department of Biostatistics, Institute of Public Health, Copenhagen University, Copenhagen, Denmark; 5 Department of Cardiology, Herlev University Hospital, Copenhagen, Denmark; Indiana University, UNITED STATES

## Abstract

**Background:**

In patients with paroxysmal atrial fibrillation (PAF) or persistent atrial fibrillation (PeAF) symptom burden and fear of hospital readmission are major causes of reduced quality of life. We attempted to develop a prediction model for future atrial fibrillation hospitalization (AFH) risk in PAF and PeAF patients including all previously experienced AFHs in the analysis, as opposed to time to first event.

**Methods:**

Recurrent event survival analysis was used to model the impact of past AFHs on the risk of future AFHs. A recurrent event was defined as a hospitalization due to a new episode of AF. Death or progression to permanent AF were included as competing risks.

**Results:**

We enrolled 174 patients with PAF or PeAF, mean follow up duration was 1279 days, and 325 AFHs were observed. Median patient age was 63.0 (IQR 52.2–68.0), 29% had PAF, and 71% were male. Highly significant predictors of future AFH risk were PeAF (HR 3.20, CI 2.01–5.11) and number of past AFHs observed (HR for 1 event: 2.97, CI 2.04–4.32, HR for ≥2 events: 7.54, CI 5.47–10.40).

**Conclusion:**

In PAF and PeAF patients, AF type and observed AFH frequency are highly significant predictors of future AFH risk. The developed model enables risk prediction in individual patients based on AFH history and baseline characteristics, utilizing all events experienced by the patient. This is the first time recurrent event survival analysis has been used in AF patients.

## Introduction

The choice of therapy in individual atrial fibrillation (AF) patients is largely dependent on AF subtype and present and predicted future symptom burden and morbidity, while the indication for anticoagulation treatment is closely related to stroke risk factors. Treatment options include rate controlling pharmacological agents, electrical and pharmacological cardioversion (CV), prophylactic antiarrhythmic medication (AAM) and catheter ablation procedures [1].

A central aspect of management decisions in patients with paroxysmal atrial fibrillation (PAF) or persistent atrial fibrillation (PeAF) is the prediction of future risk of symptomatic AF episodes, particularly if these episodes necessitate hospitalization. It is well known from both AF research and daily clinical practice that patients with more frequent and symptomatic AF episodes have an elevated risk of future AF symptoms [2,3]. Previous studies have attempted to predict future AF episodes based on wide-ranging characteristics, stretching from electrocardiography and echocardiography studies [4–10] to biomarkers and analysis of recurrence patterns in patients with implantable pacemakers or defibrillators [11–15].

### Objectives and rationale

The objective of this study was to investigate the significance of previous atrial fibrillation hospitalization (AFH) frequency and other established AF risk factors in predicting future AFHs in a cohort of PAF and PeAF patients. We assessed temporal patterns in hospitalizations using an advanced recurrent event survival analysis model which utilizes all disease recurrences in the entire study period for prediction purposes, as an alternative to studying only the time to first event. Accordingly, the objective was not to identify new previously unknown risk factors—on the contrary, we aimed to integrate known risk factors with a modern modeling approach which takes into account all events, an approach which may be more appropriate when studying a disease such as AF which is paroxysmal in nature.

The hypothesis was that past AFHs may be a strong predictor of future risk of AFHs in PAF and PeAF patients, potentially enhancing the precision of AF prediction models. Further, we hypothesized that the recurrent event survival analysis could be utilized to create a quantitative prediction model for future AFH risk in individual patients.

## Methods

### Enrollment, inclusion and exclusion

From January 1st 2008 to December 1st 2012, patients with AF were enrolled in the”Atrial Fibrillation Survey–Copenhagen (ATLAS-CPH)” from both the in- and outpatient clinics at the Department of Cardiology at University Hospital Copenhagen, Hvidovre, Denmark. Inclusion criteria were age > 18 years, recent (< 1 month) AF documented via either standard 12-lead electrocardiogram (ECG) or home monitoring, and ability to give oral and written consent. PAF was defined as at least one recorded AF episode with spontaneous conversion to sinus rhythm, no valvular AF, and excluding other temporal forms of AF. PeAF was defined as at least one recorded episode of AF lasting > 7 days, or where either medical or electrical CV was needed to restore sinus rhythm (in accordance with the Danish Cardiology Society AF guidelines at this time) [16].

Patients were excluded if AF type was permanent AF (PermAF), defined as continuous AF that was accepted by both the patient and physician, and accordingly rhythm control interventions were not pursued. Patients were also excluded if they had previously been, or were at any time during the follow up period, treated for AF with an invasive ablation procedure or antiarrhythmic surgery, or if estimated survival was < 1 year from inclusion date. Patients undergoing treatment with sodium or potassium channel blocking AAM and patients with pacemakers or implantable cardioverter defibrillators (ICDs), patients with ischemic heart disease, thyroid disease, sleep apnea or electrolyte imbalances were not excluded.

### Baseline data and variables

During enrollment, numerous baseline variables were recorded through completion of an extensive questionnaire, supplemented with data from the patient’s comprehensive digitalized medical record. The variables encompassed, but were not limited to: Age, gender, lifestyle, prophylactic AAM at baseline, and cardiac as well as non-cardiac comorbidity. Recorded comorbidities included hypertension, heart failure, heart valve disease, ischemic heart disease, thyroid disease, diabetes mellitus (DM) of any type, chronic obstructive pulmonary disease (COPD), and renal disease.

### Follow-up data and recurrent event definition

Follow-up ended on March 1st 2014, and all data regarding date and duration of hospitalizations, potential progression of AF status to PermAF, and mortality in the entire follow-up period, were obtained through the patient medical records: all written text, test results and medication were available for all patients in the entire follow-up period. The records were independently evaluated by two senior medical students and author JS. Interobserver disagreements were resolved by attainment of a consensus.

Recurrent events were defined as hospitalizations directly related to a new episode of symptomatic AF, with a severity or duration of symptoms leading to hospital contact, and ensuing admittance to the cardiology ward following evaluation by the cardiologist on duty. The AF diagnosis was confirmed by ECG, and possible electrical or pharmacological CV treatment was confirmed in the medical records. Only hospitalizations where symptomatic AF with or without CV treatment was the primary reason for admittance to the ward were classified as recurrent events. The obtained recurrent event time data served as the basis for the statistical analyses, which were performed by authors OB and TM.

There was no loss to follow-up in this dataset, i.e. censoring occurred only at the end of follow up for all patients. As a competing risk, a terminal event (TE) which precluded the occurrence of further recurrent events was introduced into the model. A TE was defined as either a) progression to PermAF, defined as the date on which patient and physician agreed on accepting the presence of permanent or very frequent recurrent AF, or b) death, in which case the date of death was available in the medical record and used as the terminal date. Additionally, all non-AF hospitalizations and visits to the outpatient clinic were scrutinized for potential progression to PermAF in the follow-up period.

### Statistical analyses

Using the survival package in R (version 3.2.0), Cox models for recurrent events were used to model the impact of covariates on the incidence of AFHs [17]. The model takes into account individual patient history using a multi-state approach with three possible states: no experience of recurrent events yet, 1 recurrent event, and 2 or more recurrent events. All transition intensities were proportional to each other, and the model included an absorbing state for the TE (PermAF or death). As advised [18], all probabilities including the ones for the competing event were analyzed. For details concerning use of multi-state models in recurrent event analysis, see for instance Amorim, Cai [19] or Andersen, Keiding [20]. Further, the multi-state approach has very recently been applied in a clinical context by the authors [21].

The model was used to assess both significant covariates for the risk of AF hospitalizations, and to predict individual risks. At any given time since AF diagnosis, it computed the probability of experiencing at least one new recurrent event in the future.

In all models, robust sandwich standard error estimates were used to adjust for multiple hospitalizations for the same patient. Missing covariates were handled by complete case analysis.

Estimates of the average number of AF hospitalizations were computed as a function of time since AF diagnosis. These estimates take into account potential loss to follow-up and TEs, and were performed separately for PAF and PeAF patients, but were not adjusted for any covariates [22].

## Results

### Population characteristics

A total of 189 patients met the inclusion criteria. Fifteen of these were excluded, 13 due to an invasive ablation procedure, and 2 due to estimated survival < 1 year from inclusion date, meaning 174 patients were enrolled in the study. All patients were of Caucasian ethnicity. Mean follow up duration was 1279 days, and the patients all contributed to a total of 222,459 person-days. Baseline characteristics selected for the statistical analysis were chosen a priori (see Table 1). No patients in our sample had thyroid or renal disease of any kind (therefore not shown). A total of 11% of patients used AAM at baseline, divided among propafenone (11 patients), flecainide (3 patients), cordarone (3 patients) and sotalol (2 patients). Out of these, 3 patients discontinued AAM during the follow-up period.

**Table 1:** Baseline characteristics.

	Specification	Value
**AF type**	Paroxysmal	50 (29%)
	Persistent	124 (71%)
**Gender**	Male	124 (71%)
	Female	50 (28%)
**Age**	Median (IQR)	63.0 (52.2–68.0)
**Alcohol**	0–5	93 (53%)
	>5	71 (40%)
	Missing values	10 (6%)
**Tobacco**	Never smoked	88 (51%)
	Ex-smoker	46 (26%)
	Current smoker	30 (17%)
	missing	10 (6%)
**Hypertension**		82 (47%)
**Heart failure**		14 (8%)
**Valvular heart disease**		12 (7%)
**Ischemic heart disease**		23 (13%)
**Diabetes**		24 (14%)
**COPD**		11 (6%)
**Antiarrythmic medication**		19 (11%)

Baseline characteristics of the patient population. Age in years (median and interquartile range), all other values are given as n (%).

### Statistical model results

There were 325 AFHs in the follow-up period, divided among 84 patients (1–17 events per patient). Most patients experienced from 1 to 7 events (this event number interval comprised 89% of all patient events). A TE was experienced by 45 patients prior to the study end date, 18 due to death, and 27 due to disease progression to PermAF. Interobserver disagreement in ECG or patient file evaluation was present in 16 hospitalizations (4,7%). Attainment of a consensus was subsequently reached, with 9 hospitalizations classified as AFH, and 7 classified as non-AFHs.

Aspects of the time-related data, and all the included baseline covariates, were tested for prognostic significance. The upper part of Table 2 presents the hazard ratios (HRs) with confidence intervals for the a priori chosen baseline covariates, and the number of recurrent events previously experienced by a patient. The final prediction model (lower part of Table 2) was obtained through a stepwise variable selection procedure at the 15% level, however, the DM covariate was omitted from the final model. This decision was based on the relatively small proportion of patients with DM in our patient sample (only 14%), and although DM is a risk factor for AF it may also contribute to a larger proportion of silent AF [23,24] that could not be ascertained in our study set-up, as we did not have access to continuous patient ECG monitoring. Prophylactic AAM use at baseline was not related to risk of future AFHs (HR 1.24, CI 0.77–2.01).

**Table 2:** Statistical model results.

**Provisional model** (all variables)	**Hazard ratio**	**Confidence interval (95%)**	**P-value**
AF type (PeAF)	3.60	2.36–5.47	<0.0001
1 recurrent event observed*	2.62	1.77–3.89	<0.0001
≥ 2 recurrent events observed*	6.48	4.39–9.56	<0.0001
Age	0.99	0.97–1.00	0.0253
Gender (female)	1.07	0.76–1.49	0.7022
Diabetes mellitus	0.49	0.21–1.13	0.0955
Hypertension	1.18	0.82–1.68	0.3742
Heart failure	1.10	0.61–1.99	0.7417
Valvular heart disease	0.81	0.51–1.30	0.3865
Ischemic heart disease	1.04	0.53–2.05	0.9002
COPD	1.36	0.82–2.27	0.2357
Alcohol (> 5 units / day)	0.79	0.55–1.14	0.209s
Antiarrhythmic medication	1.24	0.77–2.01	0.3734
**Final model** (selected variables	**Hazard ratio**	**Confidence interval (95%)**	**P-value**
AF type (PeAF)	3.20	2.01–5.11	<0.0001
1 recurrent event observed	2.97	2.04–4.32	<0.0001
≥ 2 recurrent events observed	7.54	5.47–10.40	<0.0001
Age	0.99	0.98–1.00	0.0909

Hazard ratios for AF hospitalizations with 95% confidence limits, and corresponding p-values. Provisional model includes all a priori chosen covariates. Final model includes only the chosen covariates (see text).

^*^ Hazard ratios for number of recurrent events observed are in reference to 0 recurrent events observed.

The highly significant determinants of the patients’ risk of future AFHs were AF type, with PeAF giving rise to a HR of 3.20 (CI 2.01–5.11), and a higher number of past AFHs resulting in increased risk, with HR 2.97 (CI 2.04–4.32) for 1 event, and HR 7.54 (CI 5.47–10.40) for 2 or more events (both HR’s compared with having 0 observed AFHs in the follow-up period). We did not find a further significant difference in future risk with increment above 2 events, and accordingly, number of past recurrent events was only divided into three categories (0, 1, and 2 or more events).

Age treated as a continuous variable showed a negligible protective effect with higher age, but was not significant, HR 0.99 (CI 0.98–1.00). The distribution and pattern of AFHs and the duration of hospitalizations were also tested for significance, but failed to add further predictive value when adjusted for total number of events (results not shown).

**Fig 1:**
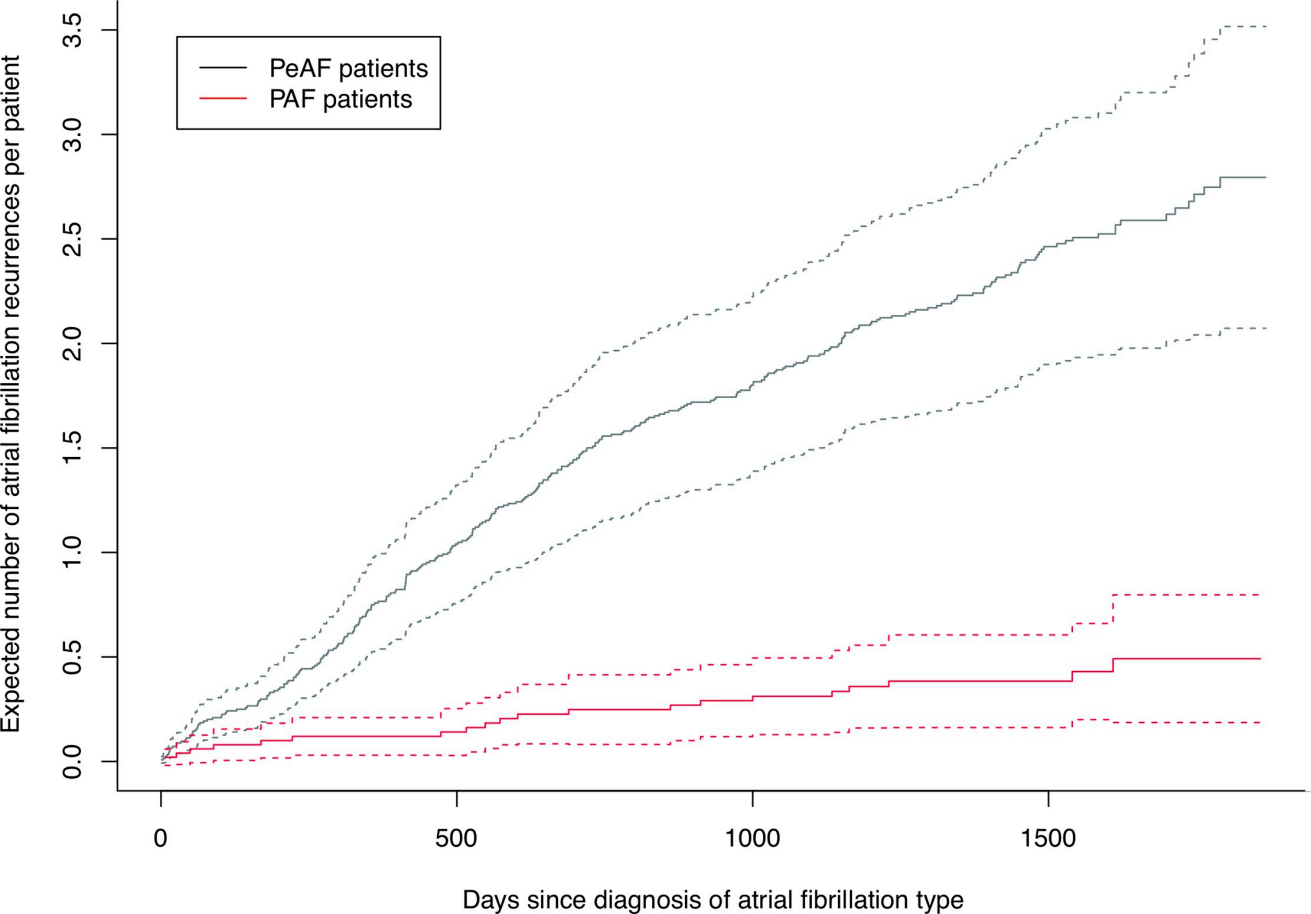
Expected number of atrial fibrillation recurrences. Comparison of risk of future AF recurrences between AF subtypes. Dotted lines represent 95% confidence intervals.

In Fig 1, the effect of AF type on the risk of future AF hospitalizations is shown, without taking into account any other covariate. There is an increased risk for PeAF patients corresponding to the increased HR shown in Table 2.

**Fig 2:**
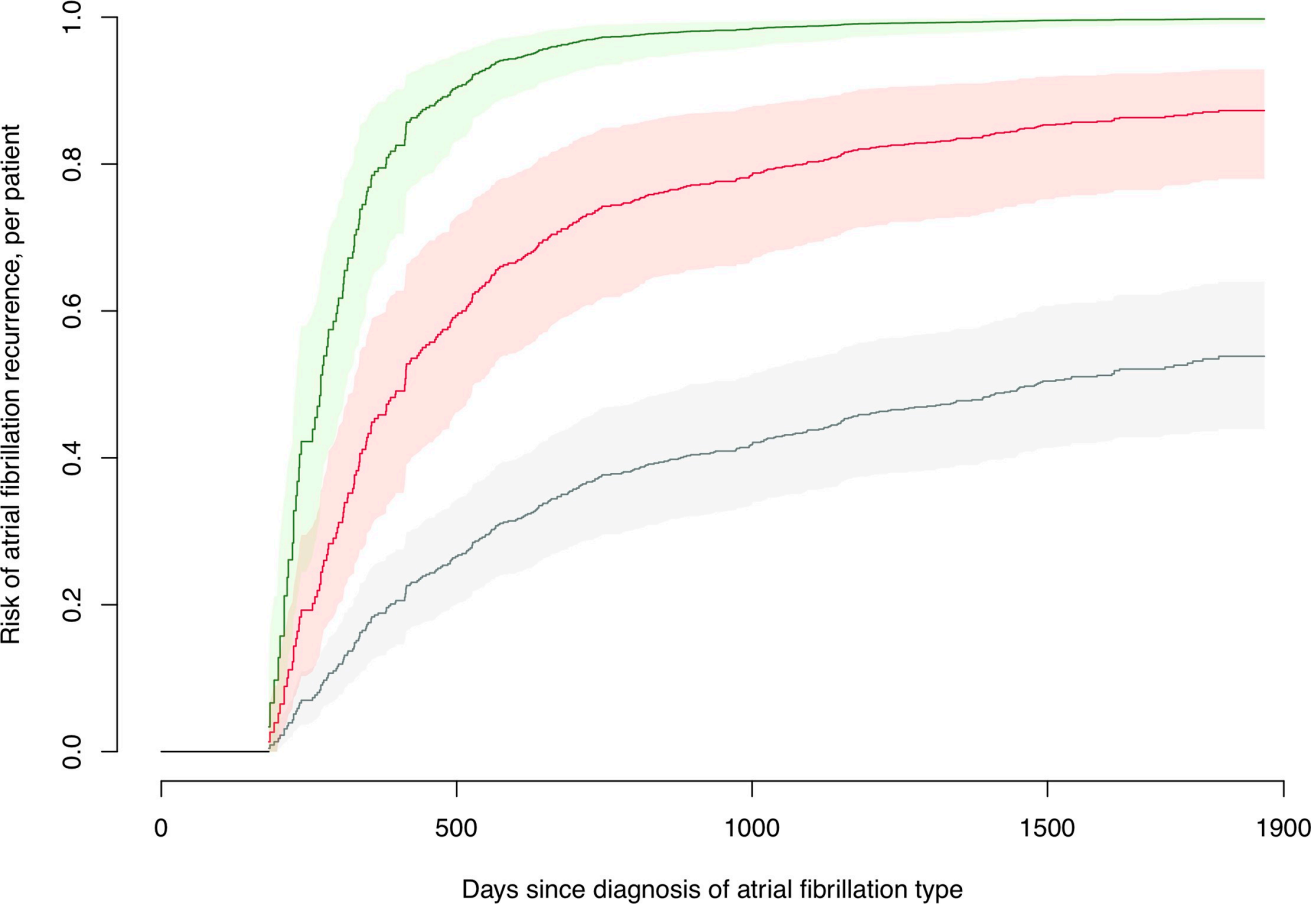
Risk of atrial fibrillation recurrence. Comparison of future AF recurrence risk for a hypothetical 60 year old patient with PeAF, 180 days after enrollment, with respectively 0 (black line), 1 (red line) or 2 or more (green line) recurrences in the last 180 days. Shadings represent 95% confidence intervals. The initial flat black line represents the 180 days in which the 0, 1, or 2 or more recurrences would have been recorded, and the risk of future events therefore starts increasing and separating between the groups from day 180 onwards.

Fig 2 exemplifies the specific risk calculation using past AFHs in a hypothetical 60 year old patient with PeAF, who is evaluated 180 days after initial AF diagnosis. Future risk of AFHs is illustrated with three curves, one for each of the three significant groups for number of past AFHs: 0 (black), 1 (red), and 2 or more (green) AFHs. Day 0 is time of AF diagnosis, and the initial 180 days on the x-axis represent the known past in which the patient may have experienced 0, 1 or 2 or more events. The future risk is then projected from day 181 onwards based on the number of AFHs experienced, i.e. if the patient had experienced 0 AFHs in the last 180 days, the risk of a new AFH in the next 500 days is predicted to be 0.27 (27%), and if the patient had experienced 1 AFH, the risk in the next 500 days is predicted to be 0.59 (59%), etc.

The competing risk of a TE only had minor impact on the risk of AFHs in the analysis, since the overall risk of a TE in our study sample was very low. This was underlined by a very negligible effect of omitting the TEs from the model and graphs (results and graphs not shown).

## Discussion

Using recurrent event survival analysis in a population of PAF and PeAF patients, our main findings were that (i) patients with PeAF generally had a significantly higher risk of future AFHs than those with PAF, (ii) in both PAF and PeAF patients, being hospitalized with AF symptoms is a major predictor of future AFHs, and (iii) the applied statistical model allows quantitative risk prediction for individual patients, taking into account both baseline characteristics and number of recurrent events in the time interval since the patient was last evaluated.

### Discussion in relation to other studies

Experimental animal studies employing artificial maintenance of AF have demonstrated that AF per se causes a greater future AF burden, and have also suggested possible pathogenetic mechanisms producing this clinically well-known phenomenon: A decrease in the threshold for inducing AF with a premature electrical stimulus, increase of the heart rate during AF, and increased stability and persistence of AF [2]. A study in AF patients has translated these findings to a clinical setting, demonstrating a prolonged disturbance in the atrial myocardium following CV for months to years after sinus rhythm has been restored [3]: In 87 patients with nonvalvular AF, atrial electrical dysfunction persisted much longer than hormonal and mechanical dysfunctions during AF. This could explain part of the vicious circle of AF recurrences in individual patients often observed in the clinical setting.

Modeling the pattern of AFHs has previously been attempted in patients with permanent implantable brady- or tachyarrhythmia pacemakers, in which case all arrhythmic events are readily accessible for analysis [25]. In 16 patients treated with dual-chamber pacing systems on bradycardia indication, AF recurrence distribution was found to be non-random and best described by clustering, and duration of AF episodes increased significantly over time [13]. In the present study, AFH distribution was not significantly related to future AFH risk when adjusted for the maintained significant predictor variables. This difference may well be explained by a combination of dissimilar modeling approaches and notable patient population differences; it is doubtful that the AF prognosis and recurrence pattern in highly selected patient subgroups with implantable pacemakers, who are often older and have more severe cardiac comorbidity, reflect the large majority of patients with PAF and PeAF which are the focus of this study.

Several smaller studies have attempted to predict future AF recurrences in patients phenotypically comparable to the cohort analyzed in the present study, based on diverse AF characteristics. In a group of 44 patients with PeAF studied with 24 hour Holter monitoring directly following CV discharge, the authors found no predictive capability with regard to AFHs at 6 month evaluation [5]. In contrast, with AFHs as our variable of interest and a mean observation time interval of over 1200 days per patient, we found a very significant HR for both AF type and number of past AFHs. Another study by Gonna et al examined 12-lead ECG p-wave duration in 77 patients following electrical CV, and found that a prolonged p-wave predicted AF recurrences, but sensitivity and specificity were relatively low (respectively 66.7% and 64.6%) [10]. Contradictory, in a related study examining P-wave characteristics in 133 AF patients the authors did not find any value of P-wave signal averaging parameters for predicting AF recurrences [9]. Thus, the precise role and potential of p-wave characteristics for AF prediction are equivocal and therefore not established at present. P-wave characteristics were regrettably not included as parameters in our study.

Other studies have utilized echocardiographic parameters for prediction of AF recurrence following direct current CV: In 95 consecutive patients with PeAF, remarkably right atrial volume index was superior to left atrial volume index in terms of predicting risk of AF at 6 months after CV, and area under the receiver operating characteristic curve was 0.77 [8]. In a different study of PeAF patients, all of which had enlarged atria (left atrial volume >34 mL/m^2^), an increased E/e’ ratio >11 indicating left-ventricular diastolic dysfunction was related to risk of AF recurrence with an odds ratio of 3.25 [26]. It may be speculated that electrocardiographic and echocardiographic parameters related to increased future risk of AF recurrences reflect different aspects of an underlying, common pathologic process in AF patients, which affects both chamber dimensions, cardiac mechanical function and ECG characteristics. However, the present study is not designed to differentiate which of the summarized diverse abnormal findings best predict AF recurrences and hospitalizations–instead it models a readily available clinical event, AFHs, irrespective of possible coincident presence of specific electro- or echocardiographic abnormalities.

Use of AAM was not a significant predictor of AFHs in the current study (HR 1.24, CI 0.77–2.01). This finding may partly be explained by the low number of patients using this medication (11%), i.e. insufficient statistical power to reach significance. The infrequent use of AAM is consistent with the general recommendations for rhythm control management of patients with PAF and PeAF in Denmark: To a large degree, cardiologists will prefer electrical CV and perhaps referral for ablation, and reserve prophylactic antiarrhythmic medication for resistant cases or very frequent AFHs [16]. An updated 2015 Cochrane Collaboration meta-analysis of randomized, patient-blinded trials of class I and III antiarrhythmic medication in AF found that these agents generally lowered the risk of AF recurrences [27]. For that reason a reduced HR for patients using antiarrhythmic medication might be expected in the current study as well. However, since our study is observational we suspect a considerable bias due to confounding by incidation: The patients with the highest risk of AF recurrences would also be the patients most likely to be treated with antiarrhythmic medication, and as a result would be at a higher risk of AFHs for the duration of the follow-up period. This effect may be responsible for cancelling out the protective effect of AAM in our model.

### Study strengths

By using recurrent event survival analysis and subsequently utilizing this data to predict the future risk of AFHs in specific AF patient subgroups, our prediction model expands on more standard multivariate survival analysis models. Past AFHs and PeAF as risk factors for future AFHs are not in themselves surprising findings: Rather, the strength of this new modeling approach is the use of all available data regarding AFHs and baseline risk factors, not just time to first event, for quantitative risk prediction in individual patients. The study is to our knowledge the first to take advantage of this in an AF patient population. If further developed this approach may represent a step forward in outcome analyses in AF, matching a paroxysmal disease to a more fitting statistical model.

### Study limitations

A standardized transthoracic echocardiography was not performed at enrollment. In addition, data concerning the relevant AF risk factors body mass index (BMI) and presence of sleep apnea were not recorded at patient enrollment, explaining why these variables could regrettably not be included in the analysis.

Patients treated with an ablation procedure were excluded, meaning that the results in this study only pertain to AF patients who obtained an acceptable degree of symptom control without an ablation procedure, or who had one or more contraindications to an ablation procedure.

Our definition of a recurrent event as an AF episode that led to hospitalization limited us from incorporating silent or less symptomatic AF episodes not resulting in hospitalization into the statistical model. This limitation prevented us from detecting all episodes of AF recurrences, meaning that the observed number of AF recurrences is an underestimation of the true number of AF recurrences experienced by the included patients. However, a very high frequency or duration of rhythm monitoring is required to obtain an acceptable degree of certainty that almost all AF episodes are indeed identified [15], and this is currently not feasible in a standard clinical setting. Choosing an AFH as the recurrent event of interest, instead of utilizing continuous home monitoring, may accordingly be more appropriate if the scope of the performed study is a potential future translation of the results to a clinical context.

### Future perspectives

The presented method of data analysis and modeling could be used in future studies as a valuable strategy for evaluating recurrent AF episodes or hospitalizations, and may improve prognostic prediction models in AF. Larger studies using recurrent event survival analysis in AF populations, capable of adjusting for additional relevant competing risk factors, are needed to further advance this analysis strategy and translate it to potential clinical implementation.

## Declarations

### Ethics

The study complies with the Declaration of Helsinki. Establishment of the database was approved by the Danish Data Protection Agency (J. No. 2007-41-1199) and the study was approved by the local Danish Standard Ethics Committee (J. No. H-C-2009-014). Written informed consent was obtained from all participants.

## Supporting information

S1 File Full anonymized dataset used for statistical analysis. (XLSX)

## Abbreviations

AAM: antiarrhythmic medication
AF: atrial fibrillation
AFH: atrial fibrillation hospitalization
COPD: chronic obstructive pulmonary disease
CV: cardioversion
DM: diabetes mellitus
ECG: electrocardiogram
HR: hazard ratio
PAF: paroxysmal atrial fibrillation
PeAF: persistent atrial fibrillation
PermAF: permanent atrial fibrillation
TE: terminal event
